# Is retinal vein occlusion associated with depression symptoms? A nationwide cohort study

**DOI:** 10.1097/MD.0000000000026937

**Published:** 2021-08-13

**Authors:** Minji Ha, Kyungdo Han, Younhea Jung, Daran Kim, Ji-Sun Paik, Kyung-Sun Na

**Affiliations:** a Department of Ophthalmology, Yeouido St. Mary's Hospital, College of Medicine, The Catholic University of Korea, Seoul, Republic of Korea; b Department of Statistics and Actuarial Science, Soongsil University, Seoul, Republic of Korea.

**Keywords:** depression, nationwide, retina, retinal vessel, RVO

## Abstract

Retinal vessels share similar anatomical and physiological characteristics with the cerebral microvasculature, and abnormal cerebral blood flow is reportedly associated with depressive disorder. However, there is limited evidence regarding the relationship between depression and the risk of retinal vein occlusion (RVO). This study aimed to investigate the association between depression and the prospective risk of RVO using nationally representative longitudinal data. This retrospective, nationwide, population-based cohort study included 9,178,222 people aged 20 years or older who underwent the Korean National Health Screening Program examination in 2009. The depression group consisted of subjects whose initial diagnoses were made between 2009 and 2010 (n = 128,700). The predictive value for RVO was analyzed using multivariate Cox proportional hazard regression models.

From the Kaplan–Meier curves, the depression group showed significantly higher RVO incidence probability, relative to the comparison group (*P* < .0001). After all confounding variables were adjusted, the hazard ratio of RVO in the depression group with or without recurrence was 1.2 (95% confidence interval [CI]: 1.076–1.338) and 1.087 (95% CI: 1.012–1.167), respectively, relative to the comparison group. This is the first nationwide, population-based, epidemiologic study that evaluated the association between depression and the risk of RVO development. The presence of depression was significantly associated with increased risks of RVO, and the recurrence of depression showed a higher RVO incidence probability.

## 1 Introduction

The word “depression” has numerous definitions ranging from a transient feeling of a flat mood, to serious clinical syndromes that can be severe, disabling, and recurrent.^[1]^ The World Health Organization reported depression as the third of 10 major diseases of humankind and predicted it to become the first in 2030.^[2]^ In Korea, the prevalence of major depressive disorder was 7.5% in males and 10.1% in females, according to the survey of domestic mental disorders conducted in 2011, which is 1.5 times higher than that recorded in 2001 and 19.6% higher than that recorded in 2006. Previous studies showed that abnormal cerebral blood flow is associated with depression.^[3]^ Notably, cerebral and retinal microvasculature vessels share similarities in terms of anatomical and functional characteristics,^[4]^ and a plausible hypothesis may be raised that retinal vasculature abnormality reflects cerebral vasculature condition. In this context, depression has been reported to accompany early retinal microvascular changes;^[4]^ depressed individuals are at an increased risk for developing cardiovascular disease and cerebrovascular disease as well.^[5,6]^ Taken together, there may be an association between retinal vein occlusion (RVO) and depression.

Over 16 million people are affected by RVO globally.^[7]^ RVO is the second most common vision-threatening retinal vascular disease.^[7]^ It is broadly classified into central RVO (CRVO) and branch RVO (BRVO). The true incidence of RVO in a population is difficult to establish, as many RVOs are silent when the condition is mild; the patient is asymptomatic and it is only detected incidentally.^[8]^ Older age is a known risk factor for RVO,^[9]^ and with the increasing life expectancy of the global population, the prevalence of the venous occlusive disease is likely to rise in the coming decades. Personal and societal costs associated with RVO and depression are estimated to be much higher than costs for normal age-matched controls.^[10]^ Furthermore, despite adequate treatment, RVO does not completely resolve because of secondary complications.^[11]^ For these reasons, there is an urgent need to identify new strategies for the diagnosis and treatment of RVO.

The exact role of microvascular disease and the development of RVO in depression is unknown. Interestingly, RVO and depression share common risk factors.^[9,12–14]^ Emerging evidence suggests that variations in retinal arteriolar and venular caliber reflect early pathophysiological processes linked to small-vessel diseases such as stroke^[15]^ and cognitive impairment.^[16]^ However, to the best of authors’ knowledge, there are no studies to date that have evaluated the association between depression and the risk of RVO development. In this nationwide cohort study, the association between depression and the risk of RVO was investigated in a Korean population using the National Health Insurance Service National Sample Cohort.

## 2 Materials and methods

### 2.1 Data source

This study involved the analysis of data culled from the Korean National Health Screening Program (NHSP) examination in 2009. In South Korea, the government has implemented an obligatory National Health Insurance system that covers 97% of the population and requires patients to pay only about 30% of their total healthcare cost. Therefore, the medical information of almost all patients in the healthcare institutions in Korea has prospectively integrated into the Korean National Health Insurance System (KNHIS) claim database.

### 2.2 Data collection

**Figure 1:**
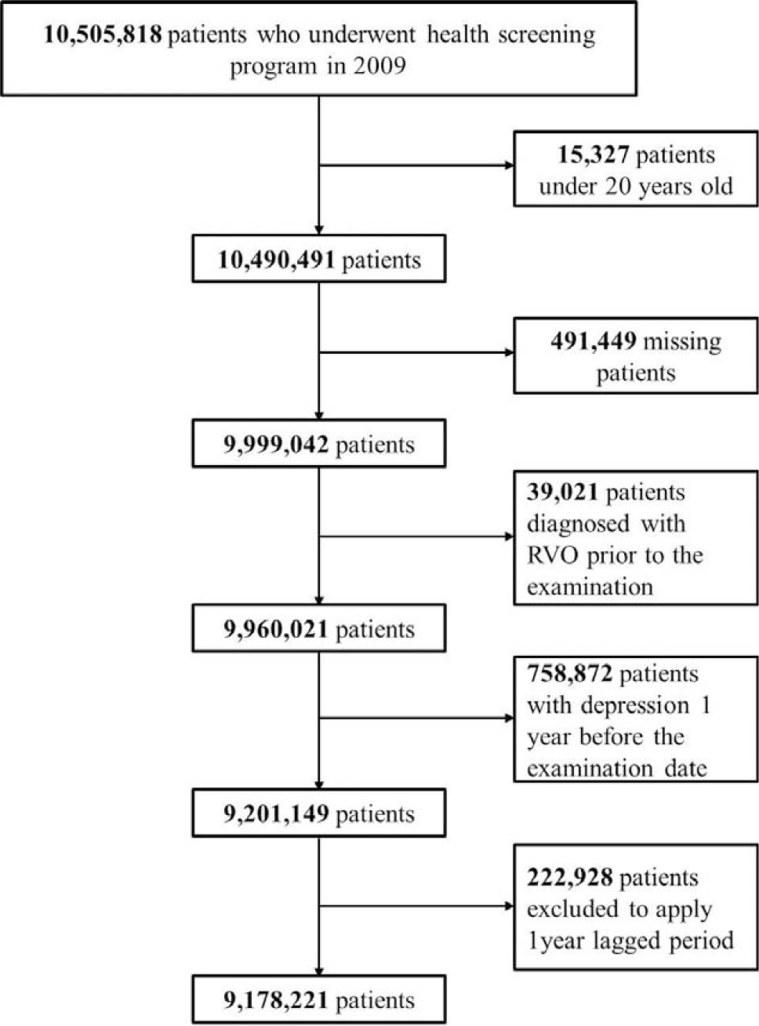
Flow diagram of sample selection for depression and RVO development analysis. RVO = retinal vein occlusion.

This study is a retrospective, nationwide, population-based cohort study. From among all individuals (n = 10,505,818) who underwent health examinations between January 1, 2009, and December 31, 2009, individuals <20 years of age (n = 15,327), those with any missing variables (n = 491,449), those with a prior diagnosis of RVO between 2002 and enrollment (n = 39,021), and those with a prior diagnosis of depression 1 year before the examination date (n = 758,872) were excluded. Additionally, 222,928 subjects were excluded to account for the 1-year lag period (Fig. 1). From the remaining 9,178,221 subjects, we identified patients who were diagnosed with depression (ICD-10 code F32, F33). Finally, 9,049,521 individuals were enrolled in the depression and comparison groups, respectively; all individuals enrolled were monitored for the development of RVO (ICD, 10th revision, clinical modification [ICD-10-CM] code H34.81, CRVO, or H34.83, venous tributary [branch] occlusion). Thus, this study included 9,178,222 people aged 20 years and older who underwent the NHSP examination at least once in 2009 and patients who were diagnosed with RVO during the washout period (2002-before healthcare check-up date). The washout period for depression was from 2002 to 1 year before the examination date and the lag period was 1 year. Follow-up was conducted until 2017. Subjects were classified into 2 groups (depression and comparison groups) based on whether they had depression, which occurred during the first year before the check-up date, or not.

### 2.3 Ethical statement

This study adhered to the tenets of the Declaration of Helsinki, and the study protocol was reviewed and approved by the Institutional Review Board of Catholic University of Medicine, Yeouido St. Mary's Hospital (CMC no. SC19ZESE0044), which waived the requirement for informed consent as well. We have been approved by the Health Insurance Corporation to use data for academic research purposes (NHIS-2019-1-576).

### 2.4 Measurements and definitions

According to the Korean NHSP examination procedures, the personal information of each patient including age, sex, the results of a questionnaire regarding past medical history and health behavior, body mass index (BMI), waist circumference, and blood pressure (BP), and results of laboratory tests such as fasting blood glucose and cholesterol level, were recorded. The diagnosis of depressive disorder was based on the diagnostic ICD-10 codes (F32-F33). Hypertension was defined as systolic BP ≥140 mmHg or a diastolic BP ≥90 mmHg or diagnosis based on ICD-10 codes (10–13, I15). Diabetes was defined as a fasting blood glucose level ≥126 mg/dL or diagnosis based on ICD-10 codes (E11–E14). Dyslipidaemia was defined as a fasting total cholesterol ≥240 mg/dL or the use of cholesterol-lowering medications or diagnosis based on ICD-10 codes (E78). BMI was calculated as body weight (kg)/height (m^2^), after measuring the height and weight of the patients in the screening test. Obesity was defined as BMI greater than 25 kg/m^2^ according to the Clinical Practice Guidelines for Overweight and Obesity compiled by the Korean Society for the Study of Obesity. Chronic kidney disease was defined as a glomerular filtration rate <60 mL/min/1.73 m^2^. Additionally, the definitions of lifestyle variables were as follows: (1) smoking status was categorized into the 3 groups namely: non-smokers, current smokers who had smoked 100 cigarettes or more in their lifetime, and ex-smokers who had smoked in the past but had quit at least 1 month ago; (2) alcohol consumption status was categorized into 3 groups namely: non-drinkers, those who drank less than 30 g of pure alcohol a day on average, and heavy drinkers who drank more than 30 g of alcohol per day. Intense exercise was defined as a fraction of 20 minutes of intense physical activity that was challenging and made breathing difficult, performed more than 3 days a week during the past week; moderate exercise was defined as the percentage of physical activity that was a bit tough on the body or made breathing slightly difficult, performed for 30 minutes at a time for more than 5 days or more per week during the past week.

### 2.5 Statistical analysis

Baseline characteristics were presented as numbers with percentages (%) for categorical variables or mean values with standard deviations for continuous variables. Chi-square test was used to analyze categorical variables and a student *t* test was used to analyze continuous variables. Kaplan–Meier survival analysis was used to draw the cumulative incidence probability of RVO. The log-rank test was used to test for differences in survival curves between the 2 groups. Associations between depression and the risk of RVO were analyzed using multivariable-adjusted Cox proportional-hazards models, and the hazard ratio (HR) and 95% confidence interval (CI) for the severity of depression, relative to the reference group (comparison group), were presented for each of the 3 models. Model 1 was adjusted for age and sex; Model 2 was further adjusted for age, sex, BMI, hypertension, diabetes, dyslipidemia, smoking, alcohol consumption; and Model 3 is an additional application of the number of visits to hospitals in Model 2. Stratified analysis was conducted for determining the association between depression and RVO development according to other characteristics of the subjects. Statistical analysis was performed with SAS version 9.4 (SAS Institute Inc., Cary, NC, USA). *P* values <.05 were considered statistically significant.

## 3 Results

### 3.1 Basic characteristics

The baseline characteristics of the study population are shown in Table 1. The subjects with depression were generally older (middle-aged), had higher BMI, larger WC, higher fasting blood glucose levels, diabetes, hypertension, dyslipidemia, were mostly non-smokers and had more females compared with the subjects in the comparison group (all *P* values <.001).

**Table 1:** Characteristics of the study population comparison group (n = 9,049,521) and depression group (n = 128,700).

Variables	Comparison group No. (%)	Depression group No. (%)
Age (yrs)		
20–39	3,017,726 (33.35%)	17,719 (13.77%)
40–64	4,976,247 (54.99%)	76,926 (59.77%)
≥65	1,055,548 (11.66%)	34,055 (26.46%)
Sex		
Male	5,133,840 (56.73%)	49,527 (38.48%)
Female	3,915,681 (43.27%)	79,173 (61.52%)
Current smoker	2,471,434 (27.31%)	20,907 (16.24%)
Drinker		
Non	8,402,821 (92.85%)	123,023 (95.59%)
Heavy	646,700 (7.15%)	5677 (4.41%)
Low income	2,382,107 (26.32%)	35,218 (27.36%)
Diabetes	742,967 (8.21%)	16,851 (13.09%)
Hypertension	2,209,781 (24.42%)	48,200 (37.45%)
Dyslipidemia	1,558,919 (17.23%)	35,618 (27.68%)
Age (years)	46.31 ± 13.91	54.59 ± 13.88
Fasting blood glucose (mg/dL)	96.9 ± 22.68	98.92 ± 24.26
BMI (kg/m^2^)	23.69 ± 3.21	23.72 ± 3.19
WC (waist circumference) (cm)	80.18 ± 9.08	80.44 ± 8.98
Systolic BP (mmHg)	122.31 ± 14.88	123.15 ± 15.46
Diastolic BP (mmHg)	76.29 ± 9.97	76.23 ± 10
GFR (mL/min/1.73 m^2^)	88.7 ± 44.73	85.48 ± 37.17
LOG (triglyceride)	113.34 (113.29–113.38)	116.35 (116–116.7)

### 3.2 Longitudinal association between depression and RVO

**Figure 2:**
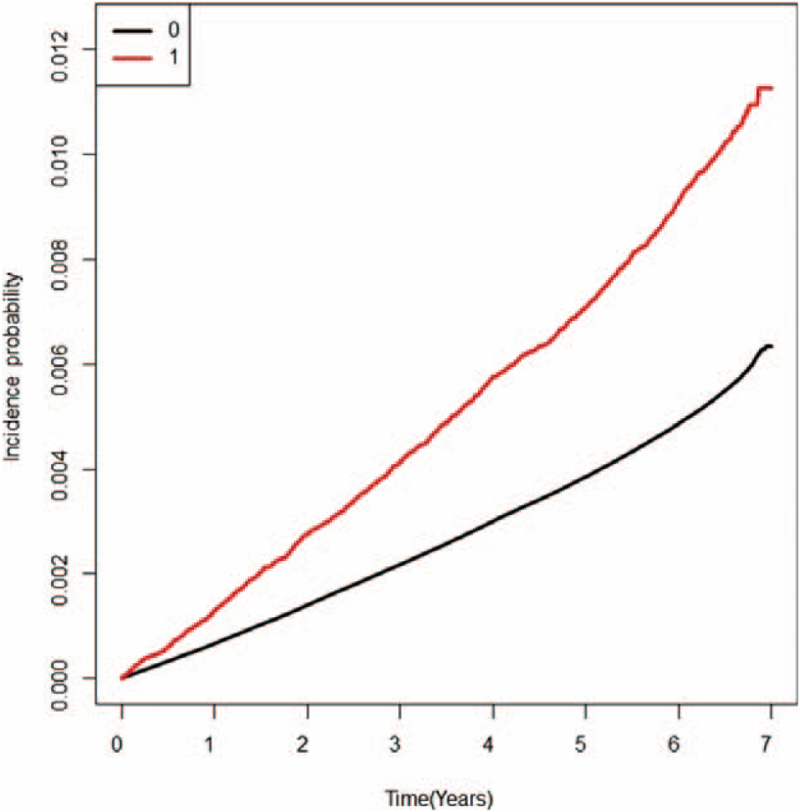
Kaplan–Meier curves showing the incidence of RVO in the depression and comparison groups. The group with depression showed a higher incidence probability than the group without depression. RVO = retinal vein occlusion.

Kaplan–Meier survival analyses were performed with RVO development as with and without depression. The log-rank test showed statistically significant *P* values <.0001. Therefore, the depression group showed a higher RVO incidence probability than the comparison group (Fig. 2). In Table 2, multivariable-adjusted Cox regression models (Models 1–3) demonstrate the influence of depression on the development of RVO in the Korean population. The adjusted HRs of RVO were 1.159 (95% CI: 1.096–1.226 for Model 1), 1.128 (95% CI: 1.066–1.193 for Model 2), and 1.118 (95% CI: 1.053–1.188 for Model 3) in the depression group compared with those in the comparison group. We further divided the severity of depression based on recurrence 1 to 2 years after the onset of depression (no recurrence group: no recurrence after 1 year from the date of onset, recurrence group: recurrence within 1 to 2 years after the date of onset; both groups had a history of depression 1 year before the examination). The adjusted HRs of RVO were 1.127 (95% CI: 1.055–1.205 for Model 1), 1.104 (95% CI: 1.033–1.18 for Model 2), and 1.087 (95% CI: 1.012–1.167 for Model 3) in the mild depression subgroup. The adjusted HRs of RVO were 1.247 (95% CI: 1.126–1.382 for Model 1), 1.192 (95% CI: 1.077–1.321 for Model 2), and 1.2(95% CI: 1.076–1.338 for Model 3) in the severe depression subgroup. In addition, the incidence of RVO was also 2 times higher in the depression group (comparison group: 0.8415, depression group: 1.56996). Table 2 shows that the greater the severity of depression, the higher the incidence rate of RVO (comparison group: 0.84155, no recurrence group: 1.43179, and recurrence group: 2.04357).

**Table 2:** Multivariable-adjusted Cox regression analysis of the association between RVO incidence and depression.

				HR (95% CI)		
Depression	N	RVO development	RATE	MODEL 1^∗^	MODEL 2^†^	MODEL 3^‡^
No	9,049,521	47,938	0.84155	1 (reference)	1 (reference)	1 (reference)
Yes	128,700	1262	1.56996	1.159 (1.096–1.226)	1.128 (1.066–1.193)	1.118 (1.053–1.188)
Severity						
No depression	9,049,521	47,938	0.84155	1 (reference)	1 (reference)	1 (reference)
No recurrence	99,420	891	1.43179	1.127 (1.055–1.205)	1.104 (1.033–1.18)	1.087 (1.012–1.167)
Recurrence	29,280	371	2.04357	1.247 (1.126–1.382)	1.192 (1.077–1.321)	1.2 (1.076–1.338)

### 3.3 Subgroup analysis on the association between depression and RVO

Stratified analysis showed the association between depression and RVO development based on various baseline characteristics of the subjects. When we evaluated the effects of several confounding factors on RVO development according to depression, diabetes and hypertension affected the association of depression with RVO development (diabetes *P* = .0329, hypertension *P* = .0036 respectively) adjusted variable for a number of visits to hospital (Table 3).

**Table 3:** Multivariable-adjusted Cox regression analysis showing the association between depression and RVO development according to other characteristics of the subjects.

Variables	HR (95% CI)	Interaction *P* value
Age (yrs)		.3382
20–39	0.801 (0.443–1.450)	
40–64	1.121 (1.029–1.22)	
≥65	1.123 (1.046–1.243)	
Sex		.408
Male	1.065 (0.959–1.182)	
Female	1.152 (1.070–1.24)	
Drinker		.2577
Non	1.125 (1.058–1.196)	
Heavy	0.906 (0.619–1.325)	
Exercise		.5717
Non	1.114 (1.030–1.205)	
Yes	1.128 (1.026–1.24)	
Smoking		.7293
No	1.126 (1.057–1.2)	
Current	1.081 (0.897–1.302)	
Obesity		.3023
No	1.125 (1.042–1.214)	
Yes	1.104 (1.100–1.218)	
Abdominal obesity		.2338
No	1.124 (1.045–1.209)	
Yes	1.103 (0.991–1.228)	
Diabetes		.0329
No	1.127 (1.052–1.207)	
Yes	1.1 (0.968–1.249)	
Dyslipidemia		.0997
No	1.14 (1.057–1.23)	
Yes	1.089 (0.985–1.204)	
Hypertension		.0036
No	1.151 (1.051–1.261)	
Yes	1.093 (1.013–1.191)	

## 4 Discussion

In this study, we found that depression was associated with a risk of RVO development after adjusting for confounding factors including age, sex, current smoking status, alcohol consumption, exercise, income, BMI, hypertension, dyslipidaemia, diabetes, and a number of visits to the hospital in sample population drawn from a nationwide population database in Korea. Our finding, therefore, adds to a small but growing body of literature that suggests that there is an association between depression and RVO disease, possibly at the level of the microvasculature. The risk of RVO was nearly doubled with more severe depression. Additionally, most subgroups, except for those with diabetes and hypertension, did not show significant associations between depression and the development of RVO. To the best of our knowledge, this is the first study to demonstrate the direct association between depression and risk of developing RVO using nationwide, population-based cohort study data in mono-ethnic Korea.

Previous studies have found an association between depression and cardiovascular and cerebrovascular diseases.^[1,17,18]^ Interestingly, several studies have also shown that depression causes early microvascular changes and retinal vessel change.^[3,19,20]^ Retinal vessels share similar anatomical, physiological, and embryological characteristics with the cerebral microvasculature. Therefore, we can assume that the strong association between RVO and depression is related to retinal vascular changes. In addition, the risk factors for depression and RVO may be similar to those that connect depression and cardiovascular and cerebral vascular disease. Nguyen and Wong showed that retinal vascular caliber is associated with different pathophysiological processes of retinopathy, and predicts the incidence of cardiovascular disease independent of standard risk factors.^[21]^ The specific pathophysiological mechanisms of the relationship between depression and RVO are not entirely understood. However, there are some plausible explanations; these include alterations in the autonomic nervous system,^[22]^ platelet receptors and function,^[23]^ coagulopathic factors such as plasminogen activator inhibitor-1 and fibrinogen, pro-inflammatory cytokines,^[24]^ endothelial function, neurohormonal factors, and genetic linkages such as with the serotonin transporter mechanism.^[25,26]^

Some population-based and case-control studies identified all the components of metabolic syndrome, including hypertension,^[27–31]^ diabetes,^[32,33]^ and dyslipidemia, as risk factors for BRVO,^[30,34]^ after adjusting for multiple potential confounding factors. However, there has been much controversy in the available literature as to whether metabolic syndrome components affect the risk of BRVO. Intriguingly, the association between depression and RVO development was statistically significant only in hypertension and diabetes groups in our study. However, we could not confirm the relationship between dyslipidemia, abdominal obesity, obesity, sex, and age, and the RVO in depression patients (Table 3). Previous studies reported that mild forms of depression are found in up to two-thirds of patients after acute myocardial infarction,^[35]^ with the major depressive disorder being found in up to 15% of cardiovascular disease patients.^[17]^ It is over 2 or 3 times than general population.^[36]^ It is clear that depression is a risk marker for the increased incidence of cardiovascular disease. The patients with depression disorder has potential behavioral risk factor such as smoking, obesity, poor diet, and poor medication adherence.^[37]^ Therefore, these factors may interact with the biological factors described above, causing cardiovascular disease and affecting vascular disease of the retina. The mechanism is not fully understood about the direct relationship between depression and RVO, but the increased RVO incidence in the presence of depression may indicate that both diseases have a common pathogenic mechanism or that there may be other pathways in the RVO etiology than previously known.

Our study has several limitations. The most noteworthy limitation is that the diagnoses of RVO, depression, or any other comorbidity were defined on the basis of ICD codes. The depression incidence may be underestimated because of some patients not seeking medical care. And there was possible under-reporting of asymptomatic RVO or of a delayed diagnosis of RVO. Second, selection bias is present. We selected medical claim-based controls who were more likely to have comorbidities compared with controls based on the general population who neither received medical care nor had a specific diagnosis. Third, there may be reverse causality, although a washout period was considered when RVO was defined. Finally, this study did not include participants of other ethnicities.

In spite of these limitations, this study is the first extremely large-scale cohort study that evaluated the influence of depression on RVO, especially among a study population of only Koreans. Our study provides the first evidence that depression is a risk factor for RVO in almost the entire Korean population, because the KNHIS database, which includes the NHSP examination records, covers the whole population of South Korean adults. It is noteworthy that because Korea is a mono-ethnic nation, there was no need to consider racial differences. Furthermore, we comprehensively analyzed coexisting illnesses and other factors, enabling adjustment for potential confounders. Therefore, we can generalize the results of this study for Koreans because the KNHIS database that was used for this study represents the majority of the Korean adult population. Additionally, for the first time, the relationship between RVO and depressive disorder can be analyzed and used as an important basic research result for future research direction.

## 5 Conclusion

In conclusion, patients with depression exhibit a significantly higher risk for RVO after adjusting for the following confounding variables: age, sex, current smoking, alcohol consumption, exercise, income, BMI, hypertension, dyslipidemia, diabetes, and the number of hospital visits. A team-based approach, including ophthalmologists, is recommended especially if the patients are experiencing a recurrence of depressive disorder.

## Author contributions

**Conceptualization:** Kyungdo Han.

**Data curation:** Kyungdo Han.

**Formal analysis:** Minji Ha.

**Investigation:** Younhea Jung.

**Methodology:** Kyungdo Han.

**Resources:** Daran Kim, Ji-Sun Paik.

**Supervision:** Kyung-sun Na.

**Writing – original draft:** Minji Ha.

**Writing – review & editing:** Minji Ha, Kyung-sun Na.

## References

[1] Hare DL Toukhsati SR Johansson P Jaarsma T. Depression and cardiovascular disease: a clinical review. Eur Heart J 2014;35:1365–72. 24282187 10.1093/eurheartj/eht462

[2] Foulks GN Bron AJ. Meibomian gland dysfunction: a clinical scheme for description, diagnosis, classification, and grading. Ocul Surf 2003;1:107–26. 17075643 10.1016/s1542-0124(12)70139-8

[3] Liao W Wang Z Zhang X. Cerebral blood flow changes in remitted early- and late-onset depression patients. Oncotarget 2017;8:76214–22. 29100305 10.18632/oncotarget.19185 PMC5652699

[4] Meier MH Gillespie NA Hansell NK. Associations between depression and anxiety symptoms and retinal vessel caliber in adolescents and young adults. Psychosom Med 2014;76:732–8. 25373892 10.1097/PSY.0000000000000117 PMC4290848

[5] Pratt LA Ford DE Crum RM Armenian HK Gallo JJ Eaton WW. Depression, psychotropic medication, and risk of myocardial infarction. Prospective data from the Baltimore ECA follow-up. Circulation 1996;94:3123–9. 8989119 10.1161/01.cir.94.12.3123

[6] Glassman AH Shapiro PA. Depression and the course of coronary artery disease. Am J Psychiatry 1998;155:04–11. 10.1176/ajp.155.1.4 9433332

[7] Rogers S McIntosh RL Cheung N. The prevalence of retinal vein occlusion: pooled data from population studies from the United States, Europe, Asia, and Australia. Ophthalmology 2010;117:313–9. e311. 20022117 10.1016/j.ophtha.2009.07.017 PMC2945292

[8] Karia N. Retinal vein occlusion: pathophysiology and treatment options. Clin Ophthalmol 2010;4:809–16. 20689798 10.2147/opth.s7631 PMC2915868

[9] Kolar P. Risk factors for central and branch retinal vein occlusion: a meta-analysis of published clinical data. J Ophthalmol 2014;2014:724780. 25009743 10.1155/2014/724780 PMC4070325

[10] Fekrat S Shea AM Hammill BG. Resource use and costs of branch and central retinal vein occlusion in the elderly. Curr Med Res Opin 2010;26:223–30. 19921963 10.1185/03007990903439046

[11] Chatziralli I Theodossiadis G Chatzirallis A Parikakis E Mitropoulos P Theodossiadis P. Ranibizumab for retinal vein occlusion: predictive factors and long-term outcomes in real-life data. Retina 2018;38:559–68. 28248827 10.1097/IAE.0000000000001579

[12] Sun C Tikellis G Klein R. Are microvascular abnormalities in the retina associated with depression symptoms? The Cardiovascular Health Study. Am J Geriatr Psychiatry 2007;15:335–43. 17384316 10.1097/01.JGP.0000247161.98311.0f

[13] Nguyen TT Wong TY Islam FM. Is depression associated with microvascular disease in patients with type 2 diabetes? Depress Anxiety 2008;25:E158–162. 17966124 10.1002/da.20427

[14] Newman-Casey PA Stem M Talwar N. Risk factors associated with developing branch retinal vein occlusion among enrollees in a United States managed care plan. Ophthalmology 2014;121:1939–48. 24953793 10.1016/j.ophtha.2014.04.045 PMC4177949

[15] Wong TY Klein R Couper DJ. Retinal microvascular abnormalities and incident stroke: the Atherosclerosis Risk in Communities Study. Lancet 2001;358:1134–40. 11597667 10.1016/S0140-6736(01)06253-5

[16] Wong TY Klein R Sharrett AR. Retinal microvascular abnormalities and cognitive impairment in middle-aged persons: the Atherosclerosis Risk in Communities Study. Stroke 2002;33:1487–92. 12052979 10.1161/01.str.0000016789.56668.43

[17] Colquhoun DM Bunker SJ Clarke DM. Screening, referral and treatment for depression in patients with coronary heart disease. Med J Aust 2013;198:483–4. 23682890 10.5694/mja13.10153

[18] Nicholson A Kuper H Hemingway H. Depression as an aetiologic and prognostic factor in coronary heart disease: a meta-analysis of 6362 events among 146 538 participants in 54 observational studies. Eur Heart J 2006;27:2763–74. 17082208 10.1093/eurheartj/ehl338

[19] Merikangas KR He JP Burstein M. Lifetime prevalence of mental disorders in U.S. adolescents: results from the National Comorbidity Survey Replication--Adolescent Supplement (NCS-A). J Am Acad Child Adolesc Psychiatry 2010;49:980–9. 20855043 10.1016/j.jaac.2010.05.017 PMC2946114

[20] Dietz LJ Matthews KA. Depressive symptoms and subclinical markers of cardiovascular disease in adolescents. J Adolesc Health 2011;48:579–84. 21575817 10.1016/j.jadohealth.2010.09.001 PMC3096828

[21] Nguyen TT Wong TY. Retinal vascular manifestations of metabolic disorders. Trends Endocrinol Metab 2006;17:262–8. 16890449 10.1016/j.tem.2006.07.006

[22] de Jonge P Mangano D Whooley MA. Differential association of cognitive and somatic depressive symptoms with heart rate variability in patients with stable coronary heart disease: findings from the Heart and Soul Study. Psychosom Med 2007;69:735–9. 17942844 10.1097/PSY.0b013e31815743ca PMC2776660

[23] Ziegelstein RC Parakh K Sakhuja A Bhat U. Depression and coronary artery disease: is there a platelet link? Mayo Clin Proc 2007;82:1366–8. 17976357 10.4065/82.11.1366

[24] Brouwers C Mommersteeg PM Nyklicek I. Positive affect dimensions and their association with inflammatory biomarkers in patients with chronic heart failure. Biol Psychol 2013;92:220–6. 23085133 10.1016/j.biopsycho.2012.10.002

[25] Parissis JT Fountoulaki K Filippatos G Adamopoulos S Paraskevaidis I Kremastinos D. Depression in coronary artery disease: novel pathophysiologic mechanisms and therapeutic implications. Int J Cardiol 2007;116:153–60. 16822560 10.1016/j.ijcard.2006.03.038

[26] de Jonge P Rosmalen JG Kema IP. Psychophysiological biomarkers explaining the association between depression and prognosis in coronary artery patients: a critical review of the literature. Neurosci Biobehav Rev 2010;35:84–90. 19962401 10.1016/j.neubiorev.2009.11.025

[27] Cheung N Klein R Wang JJ. Traditional and novel cardiovascular risk factors for retinal vein occlusion: the multiethnic study of atherosclerosis. Invest Ophthalmol Vis Sci 2008;49:4297–302. 18539932 10.1167/iovs.08-1826 PMC2584770

[28] Kawasaki R Wong TY Wang JJ Kayama T Yamashita H. Body mass index and vein occlusion. Ophthalmology 2008;115:917–8. author reply 918-919. 10.1016/j.ophtha.2007.12.002 18452768

[29] Mitchell P Smith W Chang A. Prevalence and associations of retinal vein occlusion in Australia. The Blue Mountains Eye Study. Arch Ophthalmol 1996;114:1243–7. 8859084 10.1001/archopht.1996.01100140443012

[30] Weger M Renner W Steinbrugger I. Role of thrombophilic gene polymorphisms in branch retinal vein occlusion. Ophthalmology 2005;112:1910–5. 16157382 10.1016/j.ophtha.2005.05.019

[31] Wong TY Larsen EK Klein R. Cardiovascular risk factors for retinal vein occlusion and arteriolar emboli: the Atherosclerosis Risk in Communities & Cardiovascular Health studies. Ophthalmology 2005;112:540–7. 15808241 10.1016/j.ophtha.2004.10.039

[32] Hayreh SS Zimmerman B McCarthy MJ Podhajsky P. Systemic diseases associated with various types of retinal vein occlusion. Am J Ophthalmol 2001;131:61–77. 11162981 10.1016/s0002-9394(00)00709-1

[33] Klein R Klein BE Moss SE Meuer SM. The epidemiology of retinal vein occlusion: the Beaver Dam Eye Study. Trans Am Ophthalmol Soc 2000;98:133–41. discussion 141-133. 11190017 PMC1298220

[34] Lim LL Cheung N Wang JJ. Prevalence and risk factors of retinal vein occlusion in an Asian population. Br J Ophthalmol 2008;92:1316–9. 18684751 10.1136/bjo.2008.140640

[35] Cay EL Vetter N Philip AE Dugard P. Psychological status during recovery from an acute heart attack. J Psychosom Res 1972;16:425–35. 4666660 10.1016/0022-3999(72)90068-2

[36] Kessler RC Berglund P Demler O. The epidemiology of major depressive disorder: results from the National Comorbidity Survey Replication (NCS-R). JAMA 2003;289:3095–105. 12813115 10.1001/jama.289.23.3095

[37] de Jonge P Roest AM. Depression and cardiovascular disease: the end of simple models. Br J Psychiatry 2012;201:337–8. 23118031 10.1192/bjp.bp.112.110502

